# The multisensory nature of human action imagery

**DOI:** 10.1007/s00426-022-01771-y

**Published:** 2022-11-28

**Authors:** Britta Krüger, Mathias Hegele, Martina Rieger

**Affiliations:** 1 Neuromotor Behavior Laboratory, Department of Psychology and Sport Science, Justus Liebig University Giessen, Kugelberg 62, 35394 Giessen, Germany; 2 Center for Mind, Brain and Behavior (CMBB), Philipps University of Marburg and Justus Liebig University, Giessen, Germany; 3 Institute for Psychology, UMIT Tirol-University for Health Sciences, Medical Informatics and Technology, Hall in Tyrol, Austria

## Abstract

Imagination can appeal to all our senses and may, therefore, manifest in very different qualities (e.g., visual, tactile, proprioceptive, or kinesthetic). One line of research addresses action imagery that refers to a process by which people imagine the execution of an action without actual body movements. In action imagery, visual and kinesthetic aspects of the imagined action are particularly important. However, other sensory modalities may also play a role. The purpose of the paper will be to address issues that include: (i) the creation of an action image, (ii) how the brain generates images of movements and actions, (iii) the richness and vividness of action images. We will further address possible causes that determine the sensory impression of an action image, like task specificity, instruction and experience. In the end, we will outline open questions and future directions.

## Introduction

Imagine swimming in the Caribbean Sea: In your imagination, you see the emerald waves, hear the calming sound of the breakers. You feel your arms and legs moving and feel the refreshing water around you as it gently resists your mellow movements. You taste the salty water in your mouth and smell the scents of tropical paradise around you. All of a sudden, you get pulled back to the here and now, to a cloudy autumn day, the kids arguing in a distance, you hunched over your laptop on the wooden desk you spent so much time pondering our exceptional capacity to mentally travel to distant places, to simulate or imagine sensory information that is not physically present, but still appeals to all your senses manifesting across different modalities (i.e., visual, tactile, proprioceptive, kinesthetic, auditory, olfactory and gustatory).

Mental imagery, as just described, is a high-level cognitive ability that can occur in different sensory modalities. Often, several modalities are combined supporting the notion of mental imagery as a multisensory process that uses internal representations of action and perception in working memory (Keogh & Pearson, 2017; O’Shea & Moran, 2019; Pearson, 2019). Instead of the actual execution of movements, “imaginative” perception and action are linked by prediction as a cardinal mechanism underlying mental imagery. In this regard, O'Shea and Moran (2019) argued that imagery mechanisms are an intrinsic part of the computational functioning of the brain, facilitate predictive processing (Bubic et al., 2010) and may guide future behavior (Seligman et al., 2016). There is also increasing evidence that they play an important role in both the etiology and treatment of psychopathological conditions, such as anxiety disorders, depression, or posttraumatic stress disorder (O’Shea & Moran, 2019; Reddan et al., 2018).

Consequently, numerous inquiries into the predictive architecture of the brain have indeed been concerned with the ability of humans to imagine movements and actions. In the following, we will provide an overview over the multisensory nature of imagination. More specifically, we will address the following issues: (i) the motor origin of action imagery, (ii) the brain structures involved in generating images of actions, (iii) the richness and vividness of action images that supersede a mere imagination of action outcomes. Concretely, we will address possible causes that determine the sensory impression of an action image, like task specificity, instruction and experience and imagery vividness. In the end, we will outline open questions and future directions.

### Action imagery vs. motor imagery

Motor imagery refers to the deliberate mental simulation of a movement or an action without actually executing it (Decety, 1996; Jeannerod, 1994, 2001). In the literature, traditionally the term motor imagery (e.g., Decety, 1996), rather than action imagery has been used. The term *motor* especially refers to the internal rehearsal of movements implying that the participant imagines her- or himself executing a given action or movement what inherently requires a representation of the body as the generator of acting forces, and not only of the effects of these forces on the external world (Jeannerod, 1994, 2001).

Regarding its sensory aspects, motor imagery often focuses on kinesthesis of a movement/action. Kinesthetic imagery requires one to ‘‘feel the movement’’ and to perceive muscle contractions (Roberts et al., 2008), e.g., how it feels to move the arms while swimming. In addition to kinesthetic imagery (KI), visual imagery (VI) is another major modality. Visual imagery requires visualization of a movement from a first-person perspective (internal VI), e.g., watching the arms moving ahead and to the side in front of oneself while swimming, or third-person perspective (external VI), e.g., watching the whole body, from above. The first-person perspective corresponds to the representation of a movement as if one is taking part in the action oneself; hence, suggesting that the movement is visualized as if one had a camera on one’s head. In contrast, the third-person perspective corresponds to the representation of the movement as if one was a spectator watching somebody (oneself or another person) perform the action.

However, those aspects commonly covered under the term motor imagery do not cover the whole experience during imagery of an action. First, additional sensory modalities may also be part of the imagery of actions, e.g., tasting the salt in the mouth while swimming in the sea. Second, action imagery not only includes the simulation of a movement itself, i.e., the motor part. Rather, it includes the simulation of concurrent sensations in different modalities. The sensations may be distal to the movement like the intended action effects in the environment e.g., imagery of the resulting melody while one imagines to play the piano, or they may be proximal sensory consequences of movement such as feeling the nubby surface of a basketball while practicing imaginary of free throws. Third, imagery of actions may also include aspects of the environment that are not directly related to the movement itself, but nevertheless important for it, e.g., the sound of music to which one is dancing. On the basis of these considerations, we prefer the term action imagery over the term motor imagery as it emphasizes that the imagined movement is accompanied by related and very specific sensory impressions in several modalities. Thus, it becomes necessary to expand the definition on motor imagery above, to capture what we mean by action imagery: Action imagery refers to the (deliberate) mental simulation of a movement without actually executing it. It can be experienced in several sensory modalities and may also address sensations which are rather indirectly related to the movement.

## The motor origin of action imagery

In general, our ability to engage in mental imagery manifests as a complex perception-like process in the absence of any external stimulus input (Annett, 1995; Cumming & Eaves, 2018; Farah, 1984; Kosslyn, 1987; Kosslyn et al., 2001). It involves creating the image by recalling stored sensory information. Farah (1984) proposed a computational model that describes several distinguishable components of an imagery process starting with the retrieval of information from long-term memory. In her view, information about the content of the mental image has to be passed from long-term to working memory. The retrieved image can then be transformed and maintained within working memory (see also Kosslyn, 1987, 1994). It can be inspected with the aim of detecting details to compare specific aspects with former percepts or to deliver a verbal report on imagined sensations.

Similarly, action imagery relies on experiences, which one has made with this or a similar action and which are stored in (motor) memory (Annett, 1996). Whereas early accounts suggested that action imagery is similar to planning and preparing actual action (Decety, 1996; Jeannerod, 1994), current accounts suggest that action imagery consists of a complete simulation of an action, which entails further processes related to action execution (Grush, 2004, Rieger et al., 2011, but see Glover & Baran, 2017). For example, Dahm and Rieger (2016) showed that bimanual coordination constraints during repetitive reversal movements are observed in action imagery. Further, the observation that action errors occur during action imagery indicates that action imagery includes more than planning an action, as people usually do not plan to commit errors (for an overview see Rieger et al., 2022).

In this view, action imagery comprises the ability to simulate a movement in one’s imagination, and therefore requires an internal representation of that movement, the environmental constraints and its associated sensory consequences (Munzert et al., 2009). This assumption is, among others, supported by studies using imaging techniques, which show that brain structures that contribute to the execution of actions, are active during action imagery (for a meta-analysis, see Hardwick et al., 2018).

A theoretical framework which can be used to describe the processes during action imagery is the comparator model (Davidson & Wolpert, 2005), which blossomed from a computational approach to motor control. It is based on a conception of the motor system as part of a sensorimotor loop, in which motor commands generate muscle contractions that lead to perceivable sensory feedback, which in turn influences subsequent motor commands (Wolpert & Ghahramani, 2000). Therein, the human actor is viewed as a controller that is continuously faced with noise in sensory information input and subsequent signal processing as well as considerable time delays within the sensorimotor loop. To address these issues, previous computational studies have proposed that the central nervous system (CNS) internally simulates aspects of the sensorimotor loop in planning, control and learning (for reviews, see Kawato, 1999; Wolpert, 1997). Those structures within the CNS have been termed internal models, which either mimic the input–output relationship of the controlled object (= body) or their inverses (Kawato, 1999). Accordingly, internal models come in two varieties. Forward models have a body-to-world direction of causality and predict the sensory consequences from efference copies of issued motor commands with respect to the current state of body and environment. Inverse models, on the other hand, have a world-to-body causality, specifying the necessary motor commands to bring about desired sensory consequences. By daisy- chaining the inverse and the forward model, the system can determine motor commands to achieve a certain effect in the environment through the inverse model and also compute the expected sensory feedback based on the motor commands generated by the inverse model through the forward model. Note, however, that the input to the inverse model (i.e., the intended effects of an action) and the output of the forward model (i.e., the predicted effects of an action) do not need to be identical. Culminating in the idea of neural re-use, such a domain-specific internal simulation process for controlling movements could be co-opted to facilitate information processing in a variety of non-motor domains, such as language comprehension, visual discrimination, problem solving, or mental imagery. With regard to action imagery, Kilteni et al. (2018) tested straightforward whether the strict assumption of the simulation hypothesis that action imagery engages the same mechanisms in terms of predictive computational units to generate sensorimotor predictions as real movements. Investigating the computational equivalence between motor execution and imagery, they found that imagery of an action also produces somatosensory attenuation just like real movement does underpinning the notion that action imagery engages the same central sensorimotor mechanisms.

Thus, when one intends to imagine an action, the intended action outcome is fed into the inverse models, which specifies the corresponding motor commands to execute the movement. During imagery, it is necessary to inhibit those motor commands to prevent actual movements (Guillot et al., 2012; Rieger et al., 2017), while a copy of the motor commands is sent to the forward model, which would then use this information to predict both the state of our body had we actually carried out the movement along with the sensory consequences that the movement is likely to generate (sensory) predictions (Blakemore & Sirigu, 2003; Davidson & Wolpert, 2005; Desmurget & Grafton, 2000; Grush, 2004; Johansson & Flanagan, 2009; Wolpert & Flanagan, 2001; Wolpert & Miall, 1996). One crucial aspect of the viability of a forward model as a means to compensate time delays in the online control of actions pertains to its ability to provide an internal trace of the sensory signals associated with the executed movements, a function that has been a prominent part in many theories of motor control and learning. Adams’ memory trace (Adams, 1971) as well as Schmidt’s (Schmidt, 1975) recognition schema all emphasize the continuous nature of the expected sensory consequences of motor commands that could be compared against the intended sensory consequences as the movement unfolded. Thus, it is not merely the action outcome that is predicted by a forward model, but rather the whole series of sensations that accompany an action throughout its whole execution. These sensations can of course pertain to every sensory modality as described in the introductory example. If this was true, one should be able to find evidence for multimodal representations of remote and distal sensory signals associated with movement execution that go well beyond a mere prediction of the final effects of an action as suggested by Bach et al. (2022). According to their ideomotor approach to action imagery, actions are represented by their perceivable effects, which are then translated into the motor behaviors to which they are associated. Thus, any activation of the effect image, either endogenously or exogenously, will trigger the corresponding action representation (Shin et al., 2010). Based on these ideas, Bach et al. propose that action imagery does not rely on neural re-use of execution-related motor units, but instead reflects specifically the perceptual process through which people plan and initiate their action, i.e., the action effects. Problems of ideomotor accounts arise however, with respect to explaining the influence of bodily states on imagery performance (for example, de Lange et al., 2006; Lorey et al., 2009) and the fact that action imagery pertains to the whole course of an action and not only of its outcome (Gallese & Sinigaglia, 2011; Rieger et al., 2022).

The internal action representations described above are built up by our on-going experience of our movements and its consequences in the world. Studies on action execution investigating how underlying internal representations and sensorimotor contingencies are built up have also investigated which kind of sensory information (i.e., either visual or proprioceptive) is crucial for building a well-adapted predictive forward model. Findings have suggested that sensory modalities are weighted according to their statistical significance during learning (Templeton et al., 1966; van Beers et al., 1999; Welch & Warren, 1980). For example, for tasks requiring high precision for their successful execution, like a goal-oriented throwing task, it has been demonstrated that visual signals are the crucial input to the predictive forward model (Joch et al., 2018). For music skill acquisition, however, the process of achieving skilled performance relies, besides visual and somatosensory feedback, especially on auditory feedback (Bangert et al., 2001). Thus, the relevance of visual, auditory and proprioceptive information differs depending on the action demand, and attention is focused selectively on those aspects of the sensory inflow that are most salient and valuable for motor learning what crucially determines the nature of the built internal model. One might therefore assume that, as imagery relies on experience-based representations of motor control, sensory modalities of the action image might be weighted in a similar way depending on their significance for the action. This subsequently determines the sensory qualities of the action image.

### Neural representations of action imagery

Over the past 30 years, the results of numerous studies have led to a picture of action imagery as a process deeply rooted in the human motor system using neural structures devoted to motor control to run off-line simulations of imagined actions (Gallese, 2005; Hardwick et al., 2018; Munzert et al., 2009; Svensson et al., 2008). There is consensus that the neural substrate of action imagery is organized around several core regions: the supplementary motor area (SMA), different sections of the premotor cortex (dPMC, vPMC), the primary motor cortex (M1), posterior parietal regions, such as the inferior (IPL) and the superior parietal lobe (SPL), the basal ganglia (BG), and the cerebellum (for reviews, see Lotze & Halsband, 2006; Munzert et al., 2009; for meta-analyses, see Hardwick et al., 2018; Hetu et al., 2013). With regard to the role of the SMA during action imagery, Kasess et al., (2008) showed a strong suppressive influence of the motor imagery condition on the forward connection between SMA and M1 what highlights the importance of the SMA for suppressing movements that are represented in the motor system but not to be performed.

When examining the activation pattern during the generation of a mental action image in more detail, it becomes apparent that the detected neural activation sites within the aforementioned brain regions are not stable, but rather modulated by several factors including the imagined perspective (Lorey et al., 2009; Ruby & Decety, 2001), the imagined effector (Lee et al., 2019; Lorey et al., 2014; Piefke et al., 2009; Stippich et al., 2002), the actual body position (Lorey et al., 2009; Vargas et al., 2004), the environmental requirements of a task (Lorey et al., 2010), an individual’s imagery capacity (Guillot et al., 2008; Lorey et al., 2011; Zabicki et al., 2019), imagery strategy and instructions (Guillot et al., 2009; Lorey et al., 2009), as well as motor expertise with the imagined action (Orlandi et al., 2020).

Most of the above-mentioned studies analyzed fMRI data by assessing overall group activity changes in brain regions in response to a stimulus or a cognitive task (Decety et al., 1994; Deiber et al., 1996; Ehrsson et al., 2003; Guillot et al., 2009; Lorey et al., 2014; Lotze et al., 1999; Porro et al., 1996; Stephan et al., 1995). In more recent years, multivariate pattern analysis (MVPA) in the form of pattern classification techniques (Haynes, 2011; Kamitani & Tong, 2005) or representational similarity analysis (RSA) (Kriegeskorte & Kievit, 2013; Kriegeskorte et al., 2008) were applied to investigate the representational content of neuronal population codes. Studies using MVPA and RSA in the context of action imagery research have shown that specific features of the imagery process can be decoded from population activity within the sensorimotor system. For example, it has been demonstrated that distributed neural response patterns in (pre-)motor and posterior parietal areas can be used to distinguish the execution of a movement from its imagery (Filimon et al., 2015; Park et al., 2015; Zabicki et al., 2017), the specific imagined action (i.e., different imagined hand actions) (Pilgramm et al., 2016; Zabicki et al., 2017), or the individual impression of vividness of the imagery experience (Zabicki et al., 2019). Zabicki et al (2017), for example, demonstrated that different imagined hand actions could be decoded significantly above chance from the spatial patterns of BOLD signals in premotor and posterior parietal cortices thereby elucidating the distinctiveness of the neural codes underlying imagery of different hand actions that are characterized by different task requirements. In a follow-up study, they also showed that spatial patterns of neural activity within the premotor and parietal area reflect the perceived vividness of imagined actions (Zabicki et al., 2019).

Although these studies show that specific features of an action image, like the imagined task or the experienced vividness, are forming specific neural patterns, there is still a lack of work that describes how sensory qualities of imagined actions that differ with respect to a number of action characteristics (e.g., action requirements, the effector used, action kinematics, or individual experience) as well as with respect to an individual’s motor experience might emerge through brain activity.

## Multisensory action imagery

In action imagery, the whole variety of sensory consequences of imagined actions can be of high importance. In the next section, we will compile studies that investigate possible drivers of sensory modality in action imagery. For example, we will highlight, the role of top-down modulation of action imagery by instructions. Furthermore, we will address the questions whether some actions represented more visually, auditory and others more kinesthetically and whether the sensory impression of action imagery is a function of the task itself, of individual experience culminating in expertise, and of action imagery ability. We will also highlight studies that focus on the associated signatures in the brain. As we will see below, all of those factors may influence the sensory impressions that occur during action imagery. Importantly, the different factors interact with each other so that the final modalities used during action imagery result from a complex pattern of influencing factors and may change from moment to moment.

### Top-down modulations: imagery instructions

One may instruct participants to perform action imagery in very different ways. Most often, participants are instructed to perform either kinesthetic or visual imagery. Here, the imager’s attentional focus is placed on one sensory quality at a time. Instructed imagery strategies relate specifically to the sensory modality of the imagined action: for example, kinesthetic (feeling of the movement) or visual (from a first- or third-person perspective). Neurophysiological research indicates that brain activation differs between visual and kinesthetic action imagery (Guillot et al., 2009; Kuhtz-Buschbeck et al., 2003; Lorey et al., 2009). Guillot et al. (2009) showed that occipital regions (including the primary visual area and the extrastriate cortex) as well as superior parietal regions were recruited during instructed visual imagery; whereas increased activity in the inferior parietal lobe, the ventral premotor cortex, as well as the supplementary motor area were observed during instructed kinesthetic imagery. Thus, alongside overlapping activation patterns, there are also partially distinct networks for the different imagery modalities. This is supported by a study that showed differential activation patterns depending on the imagery strategy used while imagining hand movements. When participants were instructed to imagine movements from a first-person perspective including kinesthetic feelings (as if they were performing it), they showed stronger activation in left-hemisphere sensorimotor and posterior parietal structures, especially in the inferior parietal lobe, than they did during imagery trials using a third-person perspective (as if they were watching another person performing it) (Lorey et al., 2009). Thus, both studies indicate that an instructed sensory modality of an action image is associated with a specific neural activation pattern. An instruction seems to be a viable tool to focus the inner spotlight on a specific sensory quality and triggers a simulation on the basis of its neuronal representation.

Modality instructions also play an important role in mental practice. A common assumption is that imagery practice is most effective when multiple sensory modalities are employed (Cumming & Williams, 2012). For instance, a golfer may imagine feeling the club in the hands, seeing the movement of the club, hearing the club hit the ball, and smelling the grass. In most studies, however, visual and/or kinesthetic action imagery practice is investigated, as vision and kinesthesis are the most important modalities for most actions. The type of instruction makes a difference for skill acquisition, and further, modality instruction may interact with task characteristics (Féry, 2003). In a visual-spatial drawing task performance was better after visual action imagery practice than after kinaesthetic action imagery practice (Féry, 2003, Exp. 1). However, in a bimanual coordination task participants’ performance was better after kinaesthetic action imagery practice than after visual action imagery practice (Féry, 2003, Exp. 2). This might indicate that kinaesthetic imagery is particularly suited to acquire ordering and timing of movement elements (Féry, 2003) or for tasks with a strong motor component whereas visual imagery might be suited to acquire tasks with higher environmental precision needs.

Research manipulating imagery instructions shows that participants are able to flexibly perform imagery of the same action in different ways using different sensory modalities. An alternative to instructing participants is to solely instruct the action but leave the modalities they attend to up to them. Later, participants can be asked which modalities they attended to. This provides a situation more similar to the performance of actual actions in which people are rarely instructed which modalities they should attend to (an exemption may be specific instructions when learning a new skill in sports). Studies in which imagery modalities can be freely chosen show that the preferred modality is highly task-specific, may depend on expertise and that individual differences exist. This will be outlined below.

### Bottom-up modulation: action characteristics/type of task

When participants are not provided with specific modality instructions, they presumably choose the modalities that are best suited to imagine the task at hand. Those modalities may be different and task-dependent. For instance, whereas participants spontaneously focused more on vision than on kinesthesis in a coloring task (Rieger & Massen, 2014), no significant differences between the focus on vision and kinesthesis was observed in a reaching task (Dahm & Rieger, 2016), and higher focus on kinesthesis than on vision was observed during proximal action elements of a dart throwing task (finger grip, the arm movement, and the release of the dart, Dahm & Rieger, 2019). The overall pattern of focus on modalities is thereby similar in imagination and execution of the same action, though sometimes the focus is weaker in imagination than in execution, at least for some modalities (Dahm & Rieger, 2016; Rieger & Massen, 2014). This might indicate that the imagined sensory experience may not be as complete as the actual experience, or it might be related to individual differences in imagery ability. Nevertheless, these results support the notion that the representation of different modalities may be highly task-specific in both, imagination and in execution of actions. In coloring, vision may be particularly important to monitor the progress of an action. In reaching, visual feedback from the hands might be particularly important when the hands reach into the area of the targets at the end of the movement but kinesthesis may be important for other parts of the movement. In darts, the hands do not reach the target of the action (the bullseye) and additionally, a specific position in which the hand movement ends does not exist. Therefore, kinesthetic/tactile feedback may be more important in darts than in reaching, which is consequently represented in imagery of playing darts. A recent published study investigated the sensory impression of free imagery of a broad variety of actions regarding to their action characteristics as well as the individual experience with performing the specific action (Krüger et al., 2020a). The results demonstrate that the sensory impression of the action image can be systematically explained by properties of the imagined action (e.g., the required precision and goal orientation of a movement or the required force) underpinning the presumption of a task-related tuning of the sensory focus. For example, goal-oriented actions that require a certain but also varying degree of precision to hit a target are imagined more visually than actions that can be described as more force-related or rhythmic.

The notion that the imagined action might be a driver of the sensory quality of the action image is also underpinned by training studies, which revealed that the learning of movements with different task demands is enhanced by either visual or kinesthetic imagery (Féry, 2003; Hardy & Callow, 1999; White & Hardy, 1995). Regarding the impact of task characteristics on the sensory modality, it was demonstrated that task characteristics interact with the imagery modality: In a visual-spatial drawing task performance was better after visual action imagery practice than after kinaesthetic action imagery practice (Féry, 2003, Exp. 1). In a bimanual coordination task, however, participants’ performance was better after kinaesthetic action imagery practice than after visual action imagery practice (Féry, 2003, Exp. 2).

Evidence from neurophysiological studies that investigated mental practice of different actions indicate differential activation depending on action characteristics. In a motor learning study, Krüger et al. (2020b) asked participants to learn different sequences of a manual pointing task either physically or mentally. This sequence-learning task required that participants generate an image of the target grid, the targets as well as their moving hand on the target grid. The task, therefore, is related to an external action goal and requires a certain precision for the successful execution. The results revealed strong activation sites in the posterior parietal and visual cortices while imagining the pointing task after training. These cortical sites are associated with visual rather than kinaesthetic imagery processes (cf. Guillot et al., 2009). Another study that investigated training outcomes (Lebon et al., 2018) examined associations between fMRI activation measured prior to action imagery practice and kinesthetic or visual imagery strategies and their effect on motor performance in a finger sequence task without any external target that had to be hit. Here, participants were instructed to move or to imagine moving the fingers of the right hand in a specific order without any target. They observed that especially high kinesthetic imagery vividness assessed before training (compared to visual imagery) and the IPL activation during imagery predicted high motor performance after training, thereby demonstrating the importance of high kinesthetic vividness for improving motor execution in the respective task. Both studies deliver initial indications regarding the relation between characteristics of the task (e.g., target vs. no target), imagery modality and a specific neural activation pattern. Thus, task-dependent usefulness of sensory information might also shape neural activation during imagery practice. However, a systematic investigation of such a relationship is still pending.

Most studies that investigate sensory features of action images focus on visual and kinesthetic sensory qualities. However, studies investigating performing music in one's mind and the use of imagery techniques to learn musical instruments include the acoustic modality. In general, many professional musicians use imagery techniques to rehearse various aspects of a musical piece, e.g., difficult parts of an already executed musical passage (Lotze et al., 2003). Several studies underpinning the usefulness of mental imagery in music are available on learning the trombone (Ross, 1985), piano (Bernardi et al., 2013), guitar (Theiler & Lippmann, 1995) and singing (DeSantis et al., 2021; Theiler & Lippmann, 1995). In these studies, the created action image usually includes the auditory modality (e.g., Ross, 1985). On a neurophysiological level, work that examined the singing of an Italian aria (Kleber et al., 2007) showed an involvement of the premotor cortex, SMA, and secondary auditory areas lying within the superior temporal gyrus during imagined singing. Especially the involvement of superior temporal gyrus in auditory imagery has been suggested to reflect a simulation of sound contributing to the subjective auditory experience.

In sum, all presented studies suggest that the imagined action, its characteristics and its effects mediate to a certain extent the sensory quality of the imagery process. The visual, kinesthetic and acoustic imprint are determined to a certain degree by action characteristics and are also reflected in the neural representations of the imagined actions.

### Experience and expertise

In the former sections, it became apparent that the given instruction as well as the imagined task are potent drivers for differential sensory imagery experiences. However, individual determinants such as motor expertise have also a substantial influence on the quality of the imagination process. In this vein, it has been shown that high- compared to low-level athletes reported more sensory vivid imagery (Eton et al., 1998; Isaac & Marks, 1994).

Action imagery is based on contents of (motor) memory (Annett, 1996) or internal models for the action (Davidson & Wolpert, 2005). Experts for an action should, therefore, be able to use those pre-existing internal representations for action imagery but novices should not. Several differences distinguish experts and novices. For example, hierarchically structured representations are similar between experts, but not between novices (Schack & Mechsner, 2006), indicating that expertise influences the structure of action representations. Experts in typing develop task-specific representations that are not observed in non-experts (Beilock & Holt, 2007; Rieger, 2004) and presumably have more precise internal models (Rieger, 2012), which might allow for a richer and more (sensory) detailed imagination of the action. Consequently, the duration of action imagery compared to action execution depends on expertise (Reed, 2002). Familiarity with an action may not only be relevant in actions, in which training is highly specialized, but also with simple everyday actions. When participants are asked to imagine actions, which only slightly differ from everyday actions, they may not be imagined adequately. For instance, effort applied in executed actions to compensate for additional weight may not be imagined spontaneously (Cerritelli et al., 2000; Decety et al., 1989) and familiarity with an action influences imagination durations (Rieger, 2012). One may therefore assume that differences in the imagination of different sensory modalities exist depending on experience and expertise. However, there is little research investigating this issue. Some studies indicate that the use of different modalities in imagination between experts and novices is not as different as one might think. For instance, no difference in the focus on kinesthesis and vision was found between experts and novices in imagery of dart throwing (Dahm & Rieger, 2019). Here, one explanation might be that the use of modalities in imagery is largely determined by characteristics of the task itself and not on one’s experience with it. A recent study that investigated a broad set of different actions (Krüger et al., 2020a) in a larger collective of people, however, revealed that the sensory impression of the action image can be systematically explained by a person’s idiosyncratic experience: each participant generates a more kinesthetic and a more vivid action image especially for those actions with which she or he has the most experience. In this vein, Lotze et al. (2003) investigated experienced musicians compared to amateurs during imagined violin play and demonstrated that the vividness of movement imagery was higher in the expert group and that rhythm and pitch imagination scores correlating positively with lifetime and weekly training. These results underpin the notion the sensory modality and vividness of the image is not just a matter of instruction and task, but has its roots in the imager’s individual memory that encodes for specific actions and the related sensory effects representing the individual experience with a specific action that enriches action representations.

Supporting this, a study by Fourkas et al. (2008) investigated the corticospinal excitability in forearm and hand muscles via single-pulse transcranial magnetic stimulation during mental imagery of a tennis forehand, table tennis forehand, and a golf drive in expert tennis players and novices. In tennis experts, they showed increased corticospinal facilitation during imagery of tennis, but not golf or table tennis. Corticospinal facilitation of novices was not modulated across sports. On a subjective level, the tennis experts differed only in the tennis imagery condition from novices in the ability to form proprioceptive images and to consider the tool as an extension of the hand suggesting a key role of long-term experience in modulating sensorimotor action representations that drive the (sensory) quality of the formed image.

### Individual differences in imagery ability

High imagery ability is characterized by easy generation, maintenance, and control of the imagined action. It is also associated with the subjective experience of vividness, that is, clearer, richer and entailing several sensory modalities (e.g., McAvinue & Robertson, 2008). In the nineteenth century, Galton (1880) already stated that the “detail and clarity with which individuals experience mental imagery” (p. 304) involves a difference gradient across individuals. Thus, the capacity to generate mental images is not an undifferentiated ability. The literature demonstrates variability in the individual capacity to generate action images (Cumming & Eaves, 2018; Pearson, 2019): Imagery performance differs inter- and intra-individually with respect to not only the preferred imagery perspective but also the imagery modality used (see, for a review, Moran et al., 2012). Furthermore, there are differences in imagery ability across the lifespan: young adults were significantly more accurate and rated their imagery significantly more vivid than children indicating that action imagery ability continues to develop into adulthood (Fuchs et al., 2020) and declines again in adults 70 years and older (Schott, 2012). Vividness of imagery has also a moderating effect on motor learning (e.g., Isaac, 1992): Participants who report more vivid imagery show greater performance improvements. Thus, individual differences in imagery ability relate to the effectiveness of action imagery practice (Isaac & Marks, 1994; Ruffino et al., 2017).

On the neurophysiological level, Guillot et al., (2009) compared via functional magnetic resonance imaging the pattern of cerebral activations of skilled and unskilled imagers during execution and imagery of a sequence of finger movements. Between-group comparisons revealed that participants with high imagery ability activated more the parietal and ventrolateral premotor regions, which are known to play a critical role in the generation of mental action images. By contrast, participants with low imagery abilities recruited the cerebellum, orbito-frontal and posterior cingulate cortices. The authors concluded that participants with high imagery abilities activate posterior parietal and premotor regions to a greater extent than those with low imagery abilities.

Inter-individual differences in imagery ability and imagery experience, not only in action imagery ability, relate to differences in neuronal activation pattern but also to differences in brain structure. Regarding visual imagery (not in the action context), Bergmann et al. (2016) revealed inter-individual differences in the neuronal substrate that co-varies with different aspects of the imagery experience. They observed a negative relationship between primary visual cortex (V1) surface size and sensory imagery strength, but found positive relationships between V1 surface size and imagery precision. Hence, individuals with a smaller V1 tended to have stronger, but less precise imagery. Their findings revealed the importance of V1 layout in shaping visual imagery vividness. A further study, that demonstrated inter-individual differences in imagery vividness also focused on the question whether differences in brain structure predict differences in subjective imagery vividness, here, however, in the context of auditory images. The results revealed that auditory imagery varies considerably across the investigated individuals and this variability relates to differences in the local structure of gray matter. Vividness of auditory imagery correlated with gray matter volume in the supplementary motor area (SMA), parietal cortex, medial superior frontal gyrus, and middle frontal gyrus (Lima et al., 2015). All studies underpin that brain substrate on a functional and structural level relates to the subjective ability to form rich and vivid (action) images (cf. Pearson, 2019) and highlight the role of perceptual–motor interactions for processing internally generated sensory information (cf. Lima et al., 2015; Lotze et al., 2003).

Besides inter-individual differences, imagery capacity and perceived vividness might also vary intra-individually from time to time and from image to image. Lorey et al. (2011) investigated the perceived imagery vividness during imagery of hand movements in a trial-by-trial approach with parametric functional magnetic resonance imaging. The results showed that that the perceived trial-to-trial vividness of action imagery is positively associated with neural activity within sensorimotor areas. In addition to vividness-related amplitude modulations, vividness of action images is also reflected by neural pattern in sensorimotor areas (Zabicki et al., 2019). Thus, it could be shown that similarly rated trials evoked more similar neural patterns in the left vPMC and right IPL. Furthermore, imagined actions accompanied by higher vividness ratings were significantly more distinguishable and elicited an action-specific neural pattern within the left ventral section of the premotor area as well as the right superior parietal lobe. Less vividly imagined movements, however were noisier and elicited a less action-specific noisier neural pattern, resulting in lower levels of decoding accuracy in these areas. In light of these results, the idea emerged that vividness ratings may reflect the multisensory (i.e., kinesthetic and visual) distinctiveness of an imagined action as an increased vividness rating is accompanied by a more distinct neural representation for different imagined actions. Both studies imply that functional measurements of neural activity in sensorimotor areas represent differences in subjective experience of action imagery processing. Increased vividness is accompanied by higher neural activation in sensorimotor areas (Lorey et al., 2011) but also by more distinct neural motor representation for imagined actions (Zabicki et al., 2019) reflecting the degree of success regarding the kinesthetic (and possibly visual) retrieval of action representations.

## Open questions and future research issues

Action imagery is a multisensory experience and covers the whole variety of sensory consequences of imagined actions. This is supported by the wide range of studies presented here. The studies reviewed here show that there are multiple drivers of different sensory qualities of action imagery: e.g., top-down modulations like instructions, bottom-up modulations like the imagined task, one’s idiosyncratic sensorimotor experience as well as personal characteristics like imagery ability. All of those factors influence the sensory impressions that occur during the formation of an action image and are related to a very specific signature in the brain. Action imagery relies on experience-based sensorimotor-representations built up by an individual motor learning history where the individual always uses those aspects of the sensory inflow that are most salient and valuable for task-related motor learning. This crucially determines the nature of the internal models underlying successful motor control as well as our (action) imagination.

However, despite the broad body of evidence that action imagery is accompanied and characterized by multiple sensory impressions, there are still a lot of open questions. We do not really know how the simulation process that drives action imagery—and the brain systems that support it—represent specific sensory impressions. Thus, it should be systematically investigated how sensory features of imagined actions that differ with respect to a number of characteristics (e.g., action requirements, the effector used, action kinematics, or individual experience) emerge through brain activity. We also know very little about the sensory quality of an action image when applying a bottom-up approach and investigating freely generated (uninstructed) imagery of actions that come to mind spontaneously. Here the question arises how an idiosyncratic action representation, which reflects an individual’s motor learning history, might influence the sensory impression of an action image. In this regard, it is of particular interest whether sensory features of imagined actions are deeply rooted in the action representation or formable by instruction without loss of perceived imagery quality. To understand how the sensory impression of an action image emerges on a neural level, and how it relates to the individual representation of that specific action appears to be prerequisite for understanding and developing therapeutically effective imagery interventions in the context of motor learning and re-learning, neuro-rehabilitation and psychotherapy, where decisions on how to instruct participants are so far taken on a rather intuitive basis.

It also becomes apparent that nearly all studies on action imagery focus either on visual or kinesthetic action imagery. Even though those are the most important modalities for many actions, this is not always exclusively the case. For a musician, auditory imagery may be more important than visual imagery during imagery of playing his/her own instrument. Further, even “less important” modalities may theoretically be part of an action image, e.g., the smell of nature while jogging through the forest or, as in the introductory example, the salty water while swimming in the sea. As in real actions, even though not essential for performing the action, those additional sensory features may modulate how actions are performed: the smell of nature may influence the way one breathes and tasting salt in the water or not may influence the way one holds the head while swimming. The issue of “additional” modalities has received little to no attention so far. To speculate, it may be beneficial to create multimodal images, as this may help to fine-tune specific aspects of the imagined action. If information from different modalities results in the same actions specifications, this may strengthen the specification during action imagery akin to a redundancy gain in executed actions (Miller, 1982). However, action imagery requires executive function (e.g., Glover & Barran, 2017) and multimodal action imagery may be demanding on cognitive resources. We would argue that a multimodal image is always a sign of expertise and the differentiated representation of the action. Without a well-differentiated representation via experience, multisensory imagery gets more difficult and overtaxing the system becomes more likely. We further speculate that the development of a differentiated multisensory action representation occurs particularly when motor experience was extremely rewarding, like remembering to swim in the Caribbean Sea. Indications for this can be found in the findings that show that the primary motor cortex receives dopaminergic projections from mesencephalic brainstem nuclei what is discussed to be a prerequisite of successful motor learning (Hosp & Luft, 2013).

A further issue is that in many studies the focus is on (visual or kinesthetic) imagery of the movement, only sometimes the distal effects of movements are explicitly addressed (e.g., Dahm & Rieger, 2019; Rieger & Massen, 2014). Other aspects of action imagery, most notably imagery of the environment and changes of the environment while moving in it are investigated less often. Nevertheless, those aspects of imagery may provide important insights into the mechanisms of action imagery. For instance, it has been shown that spatial updating of the own position in relation to the environment does not occur in imagined movements as it occurs in executed movement (Campos et al., 2009). Imagining the environment in which an action is usually performed might be beneficial for action imagery. The environment may act as a retrieval cue and facilitate the generation of the action image. It has already been shown that performing action imagery in an environment in which the action is usually performed is beneficial for action imagery (Guillot et al., 2005). However, so far it is unknown whether an imagined environment has similar effects.

Finally, it is unknown how do different imagined modalities interact with each other and whether similar effects as in cross-modal perception and interaction take place in multimodal imagery. One particular situation in which visual and kinesthetic/proprioceptive components sometimes contradict each other is tool use. Imagine moving a lever-like laparoscope in laparoscopic surgery. While the hand moves in one direction, the end of the laparoscope moves in the other direction. How are visual and kinesthetic/proprioceptive information represented and integrated in such a situation? Would one imagine the hand movement one executes, perhaps the touch of the laparoscope in the hand as one moves against the resisting skin and also, like X-ray vision, would one imagine seeing the tip of the laparoscope move in the tissue? To speculate, the situation might be different depending on whether one performs action imagery or action imagery practice (i.e., the systematic use of action imagery with the aim to improve future performance). In action imagery, one may attend to both, vision and kinesthesis/proprioception, or neglect one modality in favor of the other. Indeed, it has been shown that either visual or proprioceptive effect representations can be suppressed in actual tool use, depending on their relative importance for the task at hand (Liesner & Kunde, 2020). However, in action imagery practice, it may be necessary to represent the sensorimotor transformation, i.e., practicing the rule that translates body movements into movements of the tool effector. Just imaging the end of laparoscope moving without the accompanying bodily movements would probably not result in learning to perform laparoscopic surgery. Therefore, we would argue that it is necessary to represent the sensorimotor transformation, and not only the sensory effects in action imagery practice. A systematic investigation of this assumption might be a subject to future experiments. A list of open questions can be found in Table 1.

**Table 1:** List of open questions concerning the multimodal nature of action imagery

How do the simulation process and the underlying brain systems represent specific sensory impressions depending on action characteristics?
What are the sensory qualities of freely generated (uninstructed) action imagery that people engage in spontaneously?
How does an individual’s motor learning history influence the sensory impressions of an action image?
Are sensory features of imagined actions deeply rooted in the action representation or modifiable by instruction without loss of perceived imagery quality?
How can knowledge about the emergence of action images on a neural level and its relation to the individual representation of a specific action inform the development of therapeutically effective imagery interventions in the context of motor learning and re-learning, neuro-rehabilitation and psychotherapy, for instance in terms of instructions?
Which role do other modalities than visual and kinesthetic play in action imagery?
Are modalities that are non-essential for the actions part of the action image?
What are the benefits of multimodal imagery?
How do working memory capacity and executive functions related to the ability to create multimodal images?
In certain situations, is there a trade-off between different modalities?
How important is imagery of distal action effects in action imagery?
How are changes of the environment accounted for during action imagery, in particular when one changes the own position in the environment during imagery?
How do different imagined modalities interact with each other and do similar effects as in cross-modal perception take place in multimodal imagery?
Are tool actions represented as multimodal in action imagery and action imagery practice?

## Data Availability

As this article is a review article, it does not contain original data.
